# Linking a peer coach physical activity intervention for older adults to a primary care referral scheme

**DOI:** 10.1186/s12875-022-01729-4

**Published:** 2022-05-17

**Authors:** Paul L. van de Vijver, Frank H. Schalkwijk, Mattijs E. Numans, Joris P. J. Slaets, David van Bodegom

**Affiliations:** 1 Leyden Academy on Vitality and Ageing, Rijnsburgerweg 10, 2333AA Leiden, the Netherlands; 2 Department of Public Health and Primary Care, Leiden University Medical Center, Albinusdreef 2, 2333ZA Leiden, the Netherlands; 3 Department of Internal Medicine and Geriatrics, University Medical Center Groningen, Hanzeplein 1, 9713GZ Groningen, the Netherlands

**Keywords:** Peer coaching, Exercise referral, Physical activity, Primary care, Lifestyle

## Abstract

**Background:**

Physical inactivity has contributed to the current prevalence of many age-related diseases, including type 2 diabetes and cardiovascular disease. Peer coach physical activity intervention are effective in increasing long term physical activity in community dwelling older adults. Linking peer coach physical activity interventions to formal care could therefore be a promising novel method to improve health in inactive older adults to a successful long-term physical activity intervention.

**Methods:**

We evaluated the effects of linking a peer coach physical activity intervention in Leiden, The Netherlands to primary care through an exercise referral scheme from July 2018 to April 2020. Primary care practices in the neighborhoods of three existing peer coach physical activity groups were invited to refer patients to the exercise groups. Referrals were registered at the primary care practice and participation in the peer coach groups was registered by the peer coaches of the exercise groups.

**Results:**

During the study, a total of 106 patients were referred to the peer coach groups. 5.7% of patients participated at the peer coach groups and 66.7% remained participating during the 1 year follow up. The number needed to refer for 1 long term participant was 26.5. The mean frequency of participation of the referred participants was 1.2 times a week.

**Conclusion:**

Linking a peer coach physical activity intervention for older adults to a primary care referral scheme reached only a small fraction of the estimated target population. However, of the people that came to the peer coach intervention a large portion continued to participate during the entire study period. The number needed to refer to engage one older person in long term physical activity was similar to other referral schemes for lifestyle interventions. The potential benefits could be regarded proportional to the small effort needed to refer.

## Background

The proportion of older adults in the world’s population has increased and is expected to reach 2 billion in 2050 [1]. Additionally an estimated 31% of the global population does not meet the recommended level of physical activity [2]. Together, they have contributed to the current rise of age-related diseases obesity, diabetes and cardiovascular disease [2]. A diverse group of interventions to increase physical activity in healthy adults, focusing on groups or individuals, using different theoretical frameworks, including other health behavior besides physical activity, different frequency, duration and intensity of exercise sessions, using rewards or educating participants are all effective [3]. However, it is difficult to achieve sustained, long-term behavioural change after the intervention period [4–6]. It is not a feasible strategy to permanently offer costly and labour-intensive interventions to the 25% of older adults world-wide that do not achieve sufficient levels of physical activity [7]. A scalable, sustainable and affordable physical activity intervention with a large reach could be an answer to the physical inactivity challenge.

Peer coaching has been studied as a promising scalable, sustainable and affordable physical activity intervention method for older adults [8–10]. Peer coach physical activity groups are self-sustaining groups in which the training sessions are not led by professionals but by peers, people who are participants of the intervention. Our earlier research showed that this particular peer coaching intervention is a safe effective method for increasing physical activity, that the adherence to the intervention is high and that the intervention is sustainable [11–13]. Finally, peer coach groups do not depend on costly professionals and can be set up anywhere in the public space. However, peer coaching itself does not facilitate a method to involve the people that are highly likely to benefit from participating and harder to reach. Primary care practices can play an important role in advising and referring patients who are likely to benefit from increasing their physical activity. A study on the attendance in exercise programs based on an exercise referral schemes (ERS) in formal care revealed that costs, location (an intimidating gym atmosphere) and an inconvenient timing of sessions were most often reported as barriers for participation [14]. The fact that peer coach physical activity groups are low-cost, located in the public space and can take place daily are mentioned by participants as main advantages of peer coach physical activity groups [11]. Linking the community-based exercise groups to formal care might be a promising way for delivering physical exercise on a wider scale. Primary care physicians and practice nurses are in frequent contact with those aged fifty years or older and have the position and expertise to determine who is eligible for physical activity interventions. Health professionals are generally regarded as a credible source for health advice and are therefore likely to be able to influence the (un)healthy behaviors of their patients [15].

Therefore, referring patients to a peer coach physical activity intervention could be a promising addition to this successful novel method [16]. We studied the integration of a referral scheme for primary care patients to an existing peer coach physical activity intervention. We evaluated the number of referred patients by primary care professionals, adoption of referred patients and retention of referred patients in the peer coach physical activity intervention during the study period. Considering the number of people that do not meet physical activity recommendations, these efforts contribute to establishing a highly needed population wide delivery of effective, low-cost and durable strategies for increasing physical activity.

## Methods

This study evaluated the effect of an exercise referral scheme in a real-world primary care setting to a peer coach physical activity intervention. From July 2018 to April 2020 general practices actively referred patients to the peer coach groups with all practices at least referring for one year. As a comparison, from September 2016 to July 2018 eight participants joined the intervention through a ‘health care professional’, before the implementation of the ERS. However, we could not compare this number because ‘health care professional’ did also include physiotherapist and dietician. In the peer coach groups in this study, people of 50 years and older engage in an hour of peer led exercises in a public park or space in their neighbourhood on weekdays. The general and accessible fitness exercises focus on strength, flexibility, coordination and stamina [11]. A typical session starts with 5 minutes warming up by stretching the muscles and walking. Thereafter there are 20 minutes moderate intense exercises focusing on strength and stamina using weights or own bodyweight. The second part of the session is a coordination exercise of 20 minutes frequently in the form of a game using balls or other equipment. The session ends with a cooling down of 5 minutes. The groups are self-organizing and there is no monitoring from the research team. Therefore, exercise sessions vary between peer coaches and between peer coach groups. An extensive description of the format of the peer coach physical activity intervention can be found elsewhere [11, 12]. The study was registered and approved by the medical research ethics committee of Leiden University Medical Centre. All participants provided informed consent verbally.

We invited primary care practices in the vicinity of the existing peer coach physical activity groups to refer patients to the exercise groups. In the Netherlands, every citizen is enlisted with a primary care physician and more than 75% of the 50+ population sees their GP at least once a year [17]. The primary care physician acts as a gate-keeper for the access to specialized hospital care and is responsible for several primary prevention programs for chronic diseases such as cardiovascular disease and diabetes. Practice nurses, who help primary care physicians with numerous (para)medical tasks, play an important role in the delivery of these prevention programs.

The physicians and practice nurses were asked to refer patients who would potentially benefit from participating in the peer coach physical activity group. To establish a real-world setting, no further specific inclusion and exclusion criteria were imposed, as this would require more effort. The physicians and practice nurses in the participating primary care practices were asked to inform these patients on the nature of the peer coach physical activity groups and on the details of the study. They were instructed to mention the following aspects of this physical activity intervention: peer coaching, no professional, specifically for older adults, outside, in the neighbourhood, no registration and a small fee of €1 a week. The referral was not monitored. Finally, the name and date of birth of the referred patient and referral date was written down on a referral form and was given to the patient. The form contained all key aspects of the intervention; Peer coached, exercising in a group, outside, specifically for older adults, accessible for every level and participation at your own risk. Practical information consisted of exercise location and time, participation fee and that there was no need to register in advance. A carbon copy of the referral form was saved at the general practice and used to identify study participants. No other personal or medical information was retrieved as this would require a longer and more thorough informed consent conversation which would affect the referral numbers and would not be represent a realistic real-world referral.

The general practices were visited every four months to collect the forms and inform about the study progress. A referral was defined when a patient received a referral form. The participation of the referred person was recorded by daily attendance lists that were kept at the peer coach groups. Participation was defined if a person was on the attendance list. If a referred person did not attend the intervention, no further data was available. Since people are not formally enrolled in the peer coach groups, drop-out was defined as a person who did not participate for at least 3 months. A successful referral was defined as a referred person who participated once and did not dropout during the study period. Number needed to refer was calculated dividing 1 by the proportion of successful referrals. We used a Wilson score interval of the proportion successful referrals to calculate a 95% confidence interval of the number needed to refer [18]. Statistical analyses are performed with IBM SPSS Statistics for Macintosh, Version 25.0, Armonk, NY: IBM Corp.

## Results

**Fig. 1:**
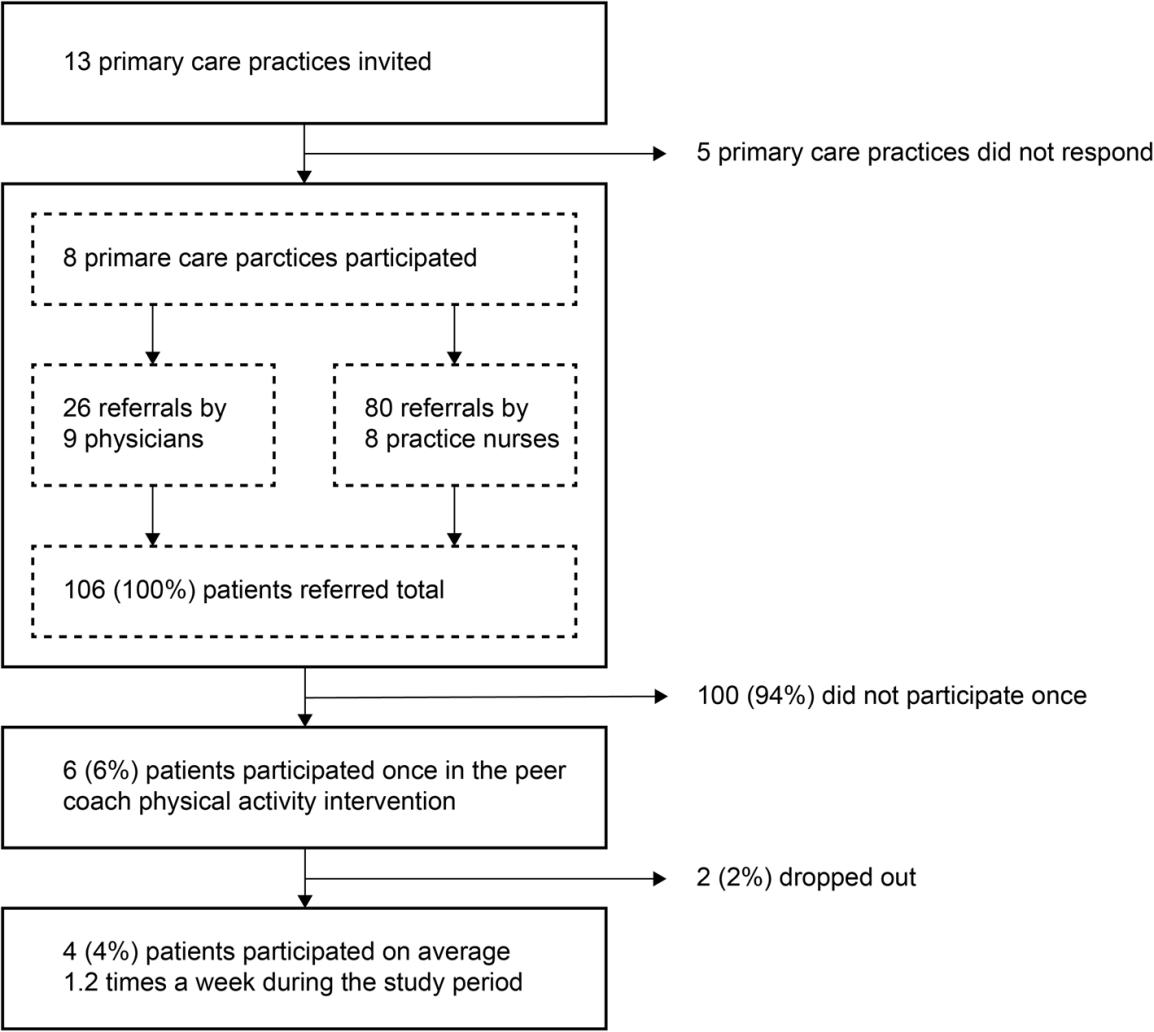
Flowchart of process evaluation of exercise referral scheme This flowchart shows the inclusion of primary care practices and referral of patients. Per participating primary care practice both the practice nurse as the physician was allowed to refer patients to the physical activity intervention

We studied the application of an exercise referral scheme for the peer coach physical activity groups in a real-world primary care setting. A summary of the inclusion of study participants and primary care practices can be seen in Fig. 1. Thirteen primary care practices located in the neighbourhood of one of the three peer coach groups were invited to participate in our study with a crude estimated total of 3250 inactive older adults. The crude estimate was derived from an average of 2000 patients per primary care, of which 1000 (50%) are aged fifty or older of which 250 (25%) do not achieve sufficient levels of physical activity. 8 practices responded positively with an estimated 2000 inactive older adults, 1 primary practice did not want to participate because they did not have the time and 4 did not react. A total of 26 older adults were referred by 9 physicians and 80 older adults were referred by 8 practice nurses, which was only 5% of the total estimated inactive older adults. 6 (6%) of the referred older adults participated in the peer coach physical activity group of which 4 (4%) continued to participate during the study period. The number needed to refer to engage one older adult in long term physical activity was 26.5 (95%CI 11–100). The median time between referral and first participation was 12 (range 1–225) days. The mean frequency of participation of the referred participants was 1.2 times a week.

Table 1 shows the number of participating physicians and practice nurses and referred patients per primary care practices in the study. 75% of referrals was done by practice nurses, whereas physicians referred 25% of the patients. More than half of all referrals were done by only one practice. Some of the participating practices referred none or only a small number of patients.

**Table 1:** Characteristics of exercise referral scheme for peer coached exercise groups, per participating primary care practice

Participating primary care practices	1	2	3	4	5	6	7	8	Total
Total referred patients*, n*	58	11	12	0	6	5	4	10	106
Referral by physicians, *n (%)*	15 (26%)	0 (0%)	4 (33%)	0 (0%)	6 (100%)	0 (0%)	1 (25%)	0 (0%)	26 (25%)
Referral by practice nurses, *n (%)*	43 (74%)	11 (100%)	8 (67%)	0 (0%)	0 (0%)	5 (100%)	3 (75%)	10 (100%)	80 (75%)
Patients that showed up at exercise group, *n (%)*	4 (7%)	2 (18%)	0 (0%)	0 (0%)	0 (0%)	0 (0%)	0 (0%)	0 (0%)	6 (6%)
Patients that remained participating during follow up, *n (%)*	2 (3%)	2 (18%)	0 (0%)	0 (0%)	0 (0%)	0 (0%)	0 (0%)	0 (0%)	4 (4%)

## Discussion

We studied the role of primary care in referring patients fifty years or older to an existing peer coach physical activity intervention. Notwithstanding the promising characteristics of peer coach activity groups as an accessible intervention for primary care patients, the success rate of the referral scheme was only 4% of the referred patients. Reviews have shown mixed evidence on the effectiveness of ERS, possibly due to the heterogeneity of ERS interventions, and the complex settings in which they take place [19–21]. Therefore, this study was performed in a real-world setting and provides a good outlook on the effect of establishing this exercise referral scheme in a Dutch primary care system.

There are several limitations and strengths to this study. The strength of this study is in the approximation of a real-world setting. No study can really imitate the real-world. However, this study approached the real-world by having no formal inclusion criteria for referring patients, having a very short informed consent procedure and no recurring contact of the physician or research team with the study participant. As a result, extensive medical and motivational information from study participants is missing. A limitation is the lack of qualitative data from professionals and patients to determine facilitators and barriers in the referral process. There is extensive research on facilitators and barriers in referral schemes [15, 22–24]. However, these studies mostly include referral schemes within the (para)medical sector [25]. Future research should examine facilitators and barriers for referral schemes to peer coach physical activity interventions.

Peer coach physical activity groups have proven to be an effective and innovative solution for increasing physical activity in a community-based setting. Previously, we have shown in a two-year follow up study that 118 people joined the exercise groups on their own initiative, and these groups continue to grow until this date [12]. There are now more than 17 peer coach physical activity groups that we know of, with more than 500 participating older adults in The Netherlands. These groups have proven to be sustainable and have a retention rate of 86% in a period of two years [12]. However, its use as part of an exercise referral scheme appears to be limited. The referred primary care patients might have been less inclined to engage in physical activity. Whereas in a community-based setting the participants have made the conscious effort themselves to join the peer coach physical activity groups, patients that were considered eligible for exercise referral have not been able or willing to find a suitable exercise opportunity.

According to the health belief model, there are various factors that are needed for health behavior change [26–28]. First, the perceived severity and susceptibility of future health problems influence to what extent a patient is inclined to engage in the ERS. Second the perceived benefits and barriers of the intervention itself play an important role. Third, a sense of self-efficacy and a cue to action are needed to make patients go to the intervention. Addressing the perceived severity and susceptibility are standard procedures in primary care, the referral serves as a cue to action. Until now the perceived barriers of costs, location (an intimidating gym atmosphere) and an inconvenient timing of sessions were most often reported as barriers for participation in exercise programs based on exercise referral schemes in formal care [14]. The major advantage of peer coaching physical activity groups is to take away these barriers. However, we hypothesize that a key characteristic of peer coaching, the fact that the sessions are not led by a health professional could have a negative effect on the perceived benefit of the intervention.

Most referrals were from one primary care practice. There are several hypotheses why the primary care professionals from this practice referred more than the others. Firstly, this was the only practice that had direct view on the exercising older adults. This visual feedback of the referrals could be a strong motivator. Secondly, the director of the primary care practice was a physician who was parttime active at the research department of the nearby academic hospital. Therefore, he had more affinity with research and this resulted in easier implementation of new programs in his practice. When interpreting these results for the real world, it is important to note that the other primary care practices more closely represent the general population of primary care practices. However, in the primary care practice with the academic director, most of the referrals were also done by a practice nurse who was similar to the practice nurses in the other primary care practices.

Another explanation for the limited effectivity of the ERS lies at the level of the health professional. Most referrals were done by practice nurses, who generally have more time to address healthy lifestyle options than primary care physicians. In a recent review, it was suggested that primary care nurses provide equal care compared to primary care doctors and that nurses achieve higher patient satisfaction levels [29]. However, it is not clear if physical activity advice from a practice nurse has the same effectivity as an advice from the primary physician. A study on the perspectives of primary care physicians on ERS emphasizes that physicians are trained to deliver pharmaceutical interventions and do not regard written exercise referrals as a priority. Physicians rather referred to other health professionals for prescribing exercise schemes [30]. Overall, physicians seem to have the least positive attitude towards preventive health interventions, compared to other health professionals [31, 32]. Most physicians rather focus on the high risk patients in their population, instead of taking a population approach to lifestyle advice [33]. However, attitudes and communication abilities of physicians remain important for achieving patient compliance in lifestyle changes. A qualitative study into the perceptions of older adults on the role of physicians in promoting physical activity showed that patients expected physical activity counselling, but that physicians did not meet these expectations [34]. Furthermore, a study into rehabilitation participation in older cardiac patients showed that the strength of physicians’ advice was the most powerful predictor for rehabilitation entry [35].

A success rate of 4% per referred patient is comparable with other lifestyle interventions in primary care [36]. The minimal intervention strategies for smoking cessation also require 33–100 referrals for one person to quit smoking [37]. And although these numbers might seem high, the large health benefits outweighs the effort. Also in this study, the cost and effort of the referral scheme are low and proportional to the time and costs of referral. Moreover, referral effectiveness can improve over time with increasing awareness of healthy lifestyle and adaptation of better referral skills by the GP’s [38]. Finally, future research on this type of minimal effort referrals must collaborate with disciplines like marketing, communication or consumer behavior. These disciplines are more experienced in recruitment and their insights can increase effectiveness of these minimal effort referrals [39, 40].

## Conclusion

Lifestyle prevention in primary care is important and an easy to implement referral scheme to an effective, durable and low-cost peer coach physical activity intervention could be an addition to counter the increasing disease burden in the worldwide ageing population. The referral scheme in this study reached only a small fraction of the target population. However, of the referred people who attended the exercise sessions, a large portion continued to exercise for a long period of time. The potential benefits could be regarded proportional to the small effort and the number needed to refer is similar to other referral schemes for lifestyle interventions.

## Data Availability

Data can be requested by contacting the corresponding author.

## Abbreviations

ERS: Exercise referral scheme
GP: General Practioner
